# Effect of the temporal coordination and volume of cyclic mechanical loading on human Achilles tendon adaptation in men

**DOI:** 10.1038/s41598-024-56840-6

**Published:** 2024-03-22

**Authors:** Meng-Shiuan Tsai, Theresa Domroes, Nikolaos Pentidis, Sophia Koschinski, Arno Schroll, Sebastian Bohm, Adamantios Arampatzis, Falk Mersmann

**Affiliations:** 1 Department of Training and Movement Sciences, Humboldt-Universität zu Berlin, Berlin, Germany; 2 Berlin School of Movement Science, Berlin, Germany

**Keywords:** Tissues, Physiology, Orthopaedics

## Abstract

Human tendons adapt to mechanical loading, yet there is little information on the effect of the temporal coordination of loading and recovery or the dose–response relationship. For this reason, we assigned adult men to either a control or intervention group. In the intervention group, the two legs were randomly assigned to one of five high-intensity Achilles tendon (AT) loading protocols (i.e., 90% maximum voluntary contraction and approximately 4.5 to 6.5% tendon strain) that were systematically modified in terms of loading frequency (i.e., sessions per week) and overall loading volume (i.e., total time under loading). Before, at mid-term (8 weeks) and after completion of the 16 weeks intervention, AT mechanical properties were determined using a combination of inverse dynamics and ultrasonography. The cross-sectional area (CSA) and length of the free AT were measured using magnetic resonance imaging pre- and post-intervention. The data analysis with a linear mixed model showed significant increases in muscle strength, rest length-normalized AT stiffness, and CSA of the free AT in the intervention group (*p* < 0.05), yet with no marked differences between protocols. No systematic effects were found considering the temporal coordination of loading and overall loading volume. In all protocols, the major changes in normalized AT stiffness occurred within the first 8 weeks and were mostly due to material rather than morphological changes. Our findings suggest that—in the range of 2.5–5 sessions per week and 180–300 s total high strain loading—the temporal coordination of loading and recovery and overall loading volume is rather secondary for tendon adaptation.

## Introduction

Tendons transmit the forces from muscles to the skeleton and are able to store and recoil elastic strain energy^1,2^. Their elasticity can facilitate the operating conditions of muscle fibers in terms of the force–length, force–velocity, power–velocity and efficiency–velocity relationship^3–5^. Therefore, the mechanical properties of tendons influence the movement performance of biological systems^6^. In humans, a high tendon stiffness seems beneficial for jumping performance, the rate of force development and electromechanical delay^7,8^. Additionally, exercise-induced increases in tendon stiffness in line with muscle strength can improve the enthalpy efficiency of the muscle and, thus, improve running economy^9^. Therefore, information on how to stimulate tendon adaptation is important for improving movement performance.

Targeted loading to increase tendon stiffness can also be essential in order to maintain a constant strain on the tendon at increasing levels of voluntary muscle force exertion. It is well established that the ultimate strain of a tendon is rather constant^10,11^. Therefore, there is commonly a strong association between muscle strength and tendon stiffness^12^. However, especially in athletes, an imbalanced muscle and tendon adaptation can lead to an increase in tendon operating strain during maximum-effort muscle contractions in children, adolescents and adults^13–16^, which is associated with an increased risk of tendon structural impairment and pain development^17,18^. Thus, a targeted increase in tendon stiffness can be beneficial for the prevention of tendon injury or to resolve the mechanical weakening of tendons commonly observed in tendinopathy^19,20^.

From a mechanobiological point of view, the magnitude, rate, duration and frequency of tendon strain determine the stimulation of the embedded tenocytes^21–23^. In a series of systematic longitudinal studies in vivo, a low frequency (i.e., 0.17 Hz) stimulation with a strain magnitude of 4.5 to 6.5% and a strain duration of about 3 s per cycle was particularly effective in triggering tendon mechanical and morphological adaptation in adults^24–26^. However, with regard to consecutive bouts of loading, it is to date unknown how the temporal coordination of loading and recovery affects the adaptation of tendon mechanical properties. In human tendons, collagen degradation and synthesis both show a significant increase following acute mechanical loading^27,28^. It has been proposed that proteolytic processes predominate the early metabolic response until protein synthesis reaches its maximum after approximately 24 h. Subsequently, positive net collagen synthesis is expected to be maintained for a further 24 h with a gradual decline afterwards^29^. Applying consecutive bouts of mechanical loading when the tissue has attained a net-anabolic state, could be a promising approach to facilitate the adaptation of tendon mechanical properties. However, to date, no studies have systematically modulated the timing of consecutive tendon loading in humans.

Another potential factor for the adaptation of biological tissues is the overall loading volume^30,31^. Also in tendons, it can be expected that within a certain range, both the acute metabolic response and long-term adaptation of tendons can be augmented with an increase in the volume of the applied mechanical load (i.e., defined here as the integral of the force-time curve). A between-study comparison of collagen synthesis rates as a function of overall loading cycles by Magnusson and colleagues^29^ suggests a non-linear dose–response relationship, which means that the increase in collagen synthesis levels off at a high cumulative load. The authors further speculate that this may initiate even a net-negative collagen balance and impair the structural properties of the tissue, which is commonly observed in overloaded tendons. Meta-analyses were not able to show systematic effects of overall loading volume on tendon adaptation in vivo^32,33^. However, this may be related to the inability of meta-analyses to identify crucial determinants of adaptation due to the heterogeneity of the included studies^34^ and to date, no study addressed the influence of loading volume experimentally.

The aim of the present study was to investigate the effects of the temporal coordination of loading and recovery and the influence of total loading volume on the time course of human tendon adaptation in young male adults. Therefore, we systematically modulated the loading frequency (in terms of sessions per week) and the weekly loading volume of an Achilles tendon (AT) loading program. To isolate the effect of these modulators, the AT loading intensity, rate and duration of single loading cycles was kept constant. Before, after eight and after 16 weeks of loading, we assessed the morphological and mechanical adaptation of the AT. We further monitored the development of tendon micromorphology for indications of structural deteriorations due to potential overload. We hypothesized that (a) repeated mechanical stimulation of the tendon at the time of estimated maximal collagen net synthesis (i.e., after 24 to 48 h of recovery) shows faster and greater adaptation effects compared to subsequent loading when the net anabolic state may already decline (i.e., after more than 48 h of recovery), and (b) that human tendons show a non-linear dose–response relationship, with greater effects of a high compared to low but not intermediate loading volume.

## Materials and methods

### Participants and experimental design

In the present study, five different AT loading protocols were applied to investigate the effects of the temporal coordination of loading and recovery and overall loading volume on AT properties. The necessary sample size of n = 12 per protocol and control group was estimated based on an a priori power analysis (performed in G*Power, Version 3.1.6; HHU, Düsseldorf, Germany) with the assumption of a large-sized time-by-protocol interaction on tendon stiffness (α = 0.05, power = 0.9, effect size *f* = 0.8, ε = 1) based on data from previous studies^24–26^. Considering that one participant could train two protocols (i.e., one per leg) and in anticipation of a potential drop-out of about 20%, 52 healthy young males aged 19 to 40 years without musculoskeletal disorders or injuries of the lower extremities within the last six months were recruited. Of all participants, 13 were assigned to the control and 39 (i.e., 78 legs) to the intervention group. Only male participants were included due to the potential effects of sex on tendon plasticity^35^. All participants signed an informed consent for the study, which was approved by the ethics committee of the Humboldt-Universität zu Berlin (No. HU-KSBFEK_2018_0017) and performed based on the guidliness formulated in the Declaration of Helsinki.

The morphology, in terms of cross-sectional area (CSA) and length of the free AT was assessed in a magnetic resonance imaging (MRI) session at baseline (week 0) and after the 16-week intervention period, while plantar flexor strength, AT mechanical properties and micromorphology were measured in weeks 0, 8, and 16. The participants were asked to refrain from strenuous physical activity for 24 h prior to the measurements. One week before the baseline measurement, all participants performed nine submaximal plantarflexion contractions with increasing effort and one maximum voluntary contraction (MVC) on the dynamometer in a familiarization session.

### Intervention protocols

Five different intervention protocols with an identical high load intensity (i.e., 90% MVC) were defined (Table 1) and performed as isometric plantarflexion contractions at ~ 0° ankle angle (i.e., sole perpendicular to the shank during the plateau of the contraction). One set consisted of four isometric plantarflexion contractions of 3 s at 90% of the MVC followed by a 3 s relaxation; there was a two-minute rest between sets. The temporal coordination of loading and recovery was modulated via the weekly loading frequency (sessions/week). The high frequency loading protocols included five sessions per week with at least 24 to a maximum of 48 h recovery between sessions (i.e., the renewed stimulus provided in the estimated phase of elevated net collagen synthesis^29^), while the low frequency loading protocols included two or three sessions per week, with at least 48 h of recovery between each session. Considering the identical intensity (i.e., percentage of MVC) in all protocols, the loading volume can be quantified as the time under loading per week (s/week) and was modulated via the number of sets per training (sets/session). The high loading volume added up to 300 s and the low loading volume to 180 s per week. The resultant four combinations were: (1) high frequency, high volume (HFHV: 5 sessions/week, 5 sets/session), (2) high frequency, low volume (HFLV: 5 sessions/week, 3 sets/session), (3) low frequency, high volume (LFHV: 2.5 sessions/week, 10 sets/session), and (4) low frequency, low volume (LFLV: 2.5 sessions/week, 6 sets/session). Additionally, the most effective protocol from previous systematic studies of our group^24–26^ served as a reference protocol (REF) in the present study and included five sets per session and four sessions per week (with a maximum of two consecutive days of training), which represents an intermediate frequency and intermediate volume of 240 s/week. Two of the five protocols were randomly assigned to the participants of the intervention group, for the left and right leg, respectively.

**Table 1:** Overview of the loading structure of the applied loading protocols.

Group	Loading frequency (sessions/week)	Duration between sessions (h)	Number of sets (sets/week)	Loading volume (s/week)
HFHV	5	24–48	5	300
HFLV	5	24–48	3	180
LFHV	2.5	48–96	10	300
LFLV	2.5	48–96	6	180
REF	4	24–72	5	240
Control	0	N/A	0	0

One set contained 4 × 3 s under loading at 90% of the isometric voluntary maximum.

HFHV, high frequency, high volume; HFLV, high frequency, low volume; LFHV, low frequency, high volume; LFLV, low frequency, low volume; REF, reference protocol; N/A, not applicable.

**Figure 1:**
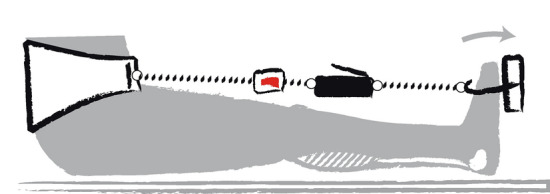
The mobile training system consisted of a kite-surfing harness, load scale, adjustable ratchet strap, and a stirrup. The scale provided instant feedback on the force applied to the stirrup during the isometric plantarflexion for the participants to adjust to the targeted intensity of the protocol. The length of the ratchet strap was adjusted so that an ankle angle of ~ 0° (i.e., sole perpendicular to the shank) was achieved during the plateau of the contraction.

**Figure 2:**
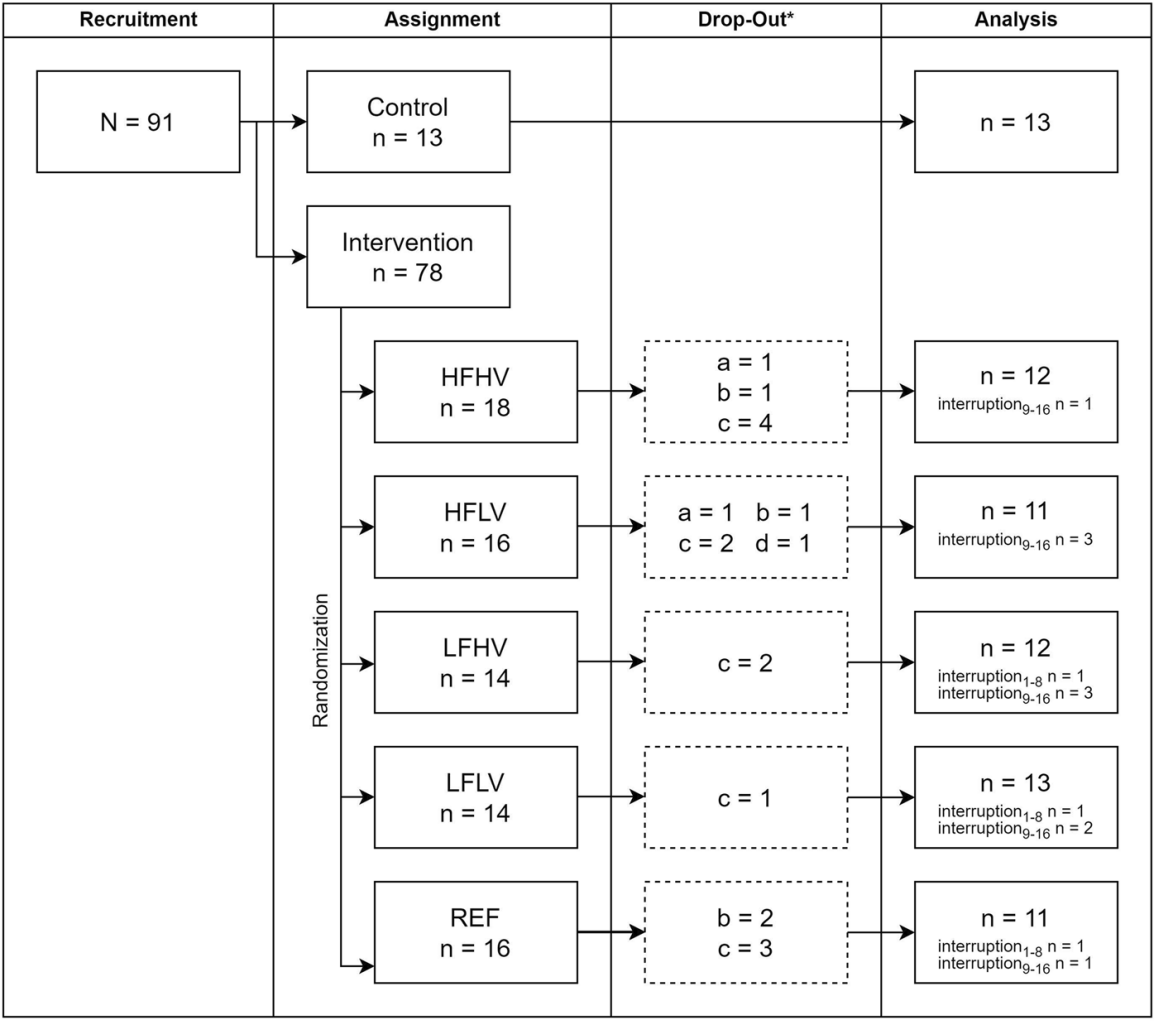
Flowchart of the process from recruitment to final analysis in the five loading intervention groups and the control group (N or n refers to the number of assigned legs, not participants). HFHV, high frequency, high volume; HFLV, high frequency, low volume; LFHV, low frequency, high volume; LFLV, low frequency, low volume; REF, reference protocol. ^*^Reasons for drop-outs: (**a**) pain in the foot arch, (**b**) injuries or illnesses unassociated with the intervention, (**c**) insufficient time or personal reasons, and (**d**) insufficient quality of the ultrasound images. interruption: number of participants who experienced a training interruption in week 1–8 or week 9–16.

To provide a gradual increase in loading in order to avoid overuse, in the first 2 weeks, all intervention protocols consisted of three sets per session, two sessions per week with 70% of the individual MVC. The subsequent week was protocol-specific in terms of frequency and number of sets, yet with only 70% MVC. The loading during the final 13 weeks was then protocol-specific with 90% MVC. The 16-week AT loading intervention was conducted using a self-developed and validated mobile home-training system^36^, to reduce physical contact during the COVID-19 pandemic. The system consisted of a kite-surfing harness connected in series to a load scale, an adjustable ratchet strap, and a stirrup (Fig. 1). The scale provided instant feedback on the force applied to the stirrup during the isometric plantarflexions, allowing the participants to control the target intensity of the protocol. In a subgroup of the participants (n = 18), AT strain was measured during maximum isometric voluntary plantarflexions in the mobile training system (at ~ 0° ankle angle) using the methodology described below. The measured AT strain reached 6.8 ± 1.3%. Therefore, we are confident that the average tendon strain achieved during the loading program with 90% MVC was within the range that has been reported to be an effective mechanical stimulus for the adaptation of human tendon mechanical properties in vivo^25^. Before every training session, the participants were instructed to perform a self-selected warm-up that induced low cyclic strains of the AT, such as three minutes of jogging, moderate intensity hopping or ten repetitions of submaximal plantarflexion contractions. The MVC values were independently updated by the participants every 2 weeks using the maximum of three trials in the mobile training system. The compliance with the assigned loading program was documented by the participants and was on average 94.8 ± 0.1% of the target volume and not significantly different between protocols (*p* > 0.57). An interruption for any reason of more than 2 weeks was considered a drop-out. A pause of 1 week was considered with a respective postponement of the following measurement(s). Most of these cases (10/13) occurred between week 8 and 16 (Fig. 2).

In the control group, the participants were asked to continue their habitual physical activity over the 16 weeks. For all participants, the regular physical activity of the past six months was documented in terms of average weekly hours of vigorous physical activity. There was a 20.9% drop-out (of the included total of 91 legs; 13 for the control, 78 for the intervention group, respectively) that was due to pain in the foot arch (2.2%), injuries or illnesses unassociated with the intervention (4.4%), insufficient time or personal reasons (13.2%), and insufficient quality of the ultrasound images (1.1%; Fig. 2). Seven of the included participants only contributed with data obtained either after 8 or 16 weeks (due to scheduling difficulties or insufficient ultrasound image quality).

### Assessment of muscle strength

To measure plantar flexor muscle strength, the participants performed MVCs on a dynamometer (Biodex System III; Biodex Medical Systems Inc., Shirley, NY, USA) after a standardized warm-up, which consisted of three minutes of running at 8–10 km/h and ten submaximal isometric contractions. The participants sat in a position of 100 to 110° hip flexion (supine corresponds to 180°) and full knee extension and performed isometric plantarflexion MVCs in four joint angles, equally distributed from neutral position (i.e., 0°) to the individual maximum dorsiflexion angle. There were at least two minutes of rest between the trials to avoid fatigue. Resultant ankle joint moments were calculated using an established inverse dynamics approach^37^. All kinematic data were recorded by an infrared motion capture system (Vicon Nexus, version 1.7.1., Vicon Motion System, Oxford, UK) with nine cameras operating at 250 Hz. Twelve reflective markers were fixed on the following anatomical landmarks and the dynamometer: lateral and medial femoral epicondyles and malleoli, calcaneal tuberosity, midpoint of the second and third ray of metatarsals, midpoint between greater trochanter and lateral femoral condyle, axis and lever of the dynamometer and three markers on the foot plate. To calculate the contribution of the gravitational forces, three passive (i.e., inactive muscle) dorsiflexion trials were recorded from 30° of plantarflexion to the individual maximal dorsiflexion at a speed of 5°/s. In order to consider the contribution of the antagonist muscles to the resultant ankle joint moment during maximum plantarflexions, the electromyographic (EMG) activity of the tibialis anterior was recorded using a wireless system operating at a sampling frequency of 1000 Hz (Myon m320RX, Myon AG, Baar, Switzerland). Two trials of submaximal ankle dorsiflexion contractions at the terminal angle of the maximal MVC trial were used to establish an EMG amplitude-dorsiflexion moment relationship and estimate the contribution of the antagonists during the plantarflexion MVCs^38^. The sum of the resultant ankle joint moment and the estimated antagonist moment is, henceforth, referred to as *plantarflexion moment*, and served as a measure of muscle strength.

### Mechanical properties of the Achilles tendon

The force–elongation relationship of the AT was established for the assessment of tendon mechanical properties. For this purpose, the participants performed five ramped MVCs in the same experimental setting as for the measurement of muscle strength, and the same considerations for the joint moment calculations were used. A B-mode ultrasound system (MyLab 60, Esaote, Genoa, Italy) with a 10 cm linear transducer (LA923, 7.5 MHz) was used to capture the displacement of the distal gastrocnemius medialis (GM) myotendinous junction (MTJ) during the contractions with a frequency of 25 Hz. The probe was embedded in a customized mold and attached with Velcro straps above the MTJ aligned in the direction of GM contraction. A thin sound-absorbing marker visible in the field of view of the ultrasound was placed between the skin and the probe, to be able to (a) quantify the distance (as a curved path over the skin) from the AT insertion at the tuber calcanei to the visualized aspect of the MTJ at 20° plantarflexion as *AT rest length*^39^ and (b) account for potential probe-to-skin movements. For the ramp contractions, the ankle angle was set to the neutral position (i.e., 0°) and the participants were instructed to gradually exert the force from rest to maximum effort in 5 s, guided by a customized real-time visual feedback of the moment measured at the dynamometer (interface developed in MATLAB R2016a; The MathWorks, Natick, MA, USA). Two voltage peak trigger signals were generated manually at the beginning and the end of each trial to synchronize the ultrasound images and the analog data stream to the Vicon Motion System. Five ramped MVC trials were performed and the force–elongation curves averaged to achieve a high reliability of the tendon elongation measurement^40^. The displacement of the GM-MTJ was tracked manually frame by frame over the whole contraction with a customized MATLAB script, with the rater blinded with regard to the protocol-allocation of the dataset. The displacement of the MTJ during three passive trials at 5°/s, ranging from 30° of plantarflexion to maximum dorsiflexion, was also recorded and tracked, to a) account for the MTJ displacement during the isometric contractions that is due to angular rotation and b) to estimate the AT lever arm, which was determined by applying the tendon excursion method^41,42^. The change of the lever arm during contraction was considered using the factor reported by Maganaris et al.^43^. The force applied to the AT during contraction—referred to as *AT force*—was calculated by dividing the plantarflexion moment (calculated as described above) by the tendon lever arm. For each individual, the average lever arm of all three measurement time-points was used to achieve a higher robustness in the calculation of the AT force against deviations in the probe positioning over the MTJ. The force–elongation curves from the five ramp trials were averaged up to the highest common force level and tendon stiffness was calculated with a linear regression of the averaged force–elongation data between 50 to 100% of the maximum common force during the ramp contractions. Since tendon stiffness is influenced by tendon rest length^44^, normalized AT stiffness was calculated as the product of stiffness and rest length. Tendon strain was defined as the ratio of elongation to tendon rest length multiplied by 100. Further, the maximum AT force was calculated based on the maximum plantarflexion moments during the MVC assessment, maximum tendon stress by dividing maximum AT force by the average free AT CSA (see below).

### Morphology of the Achilles tendon

Free AT length and CSA were determined by means of T1-weighted MRI. The transverse and sagittal scans (3D HYCE (GR) sequence, TR 10 ms, TE 5 ms, flip angle 80°, slice thickness 3.1 mm, 1 excitation) were captured by a 0.25 Tesla scanner (G-scan, Esaote, Genoa, Italy). The participants laid supine with the hip and knee extended, while the ankle was fixed at the neutral position (0°). The free AT CSA between the soleus muscle-AT junction (origin) and the initial attachment at the calcaneus bone (insertion) was digitalized in each transverse slice with Osirix MD (Version 10.0.3, Pixmeo SARL, Bernex, Switzerland) by three blinded examiners using the National Institute of Health (NIH) color scale to enhance the accuracy^45^. The centroids of the CSA in each slice were determined by means of Delaunay triangulation^46^. The length of the free AT was calculated as the curved path through the centroids of the CSAs. The average CSA was the mean of all CSAs along the length of the free AT.

### Assessment of tendon micromorphology

To detect early indications for overuse-related structural changes of the tendon in the intervention group, AT micromorphology was assessed based on a spatial frequency analysis of ultrasound images^47^. The assessment was conducted prior to the warm-up in all ultrasound measurement sessions. The participants were positioned prone with the knee fully extended and the ankle fixed at 0°. Origin and insertion of the free AT were detected with a 5-cm linear transducer of an ultrasound system (My Lab60; Esaote; LA523, 13 MHz, depth: 3.0 cm) placed over the AT parallel to its longitudinal axis. Sound-absorbing rubber bands were used to mark and divide the free AT evenly into three (proximal, middle, distal) parts. Two short sequences of the distal part and the middle part were captured respectively. For image processing, two polygonal regions of interest (ROI) were defined in a custom-written MATLAB interface (version R2016b; MathWorks), with the length corresponding to one-third of the free AT of each individual and its height covering the full thickness of the tendon. Within each selected ROI, all possible kernels of 32 × 32 pixels (allowed to overlap) were extracted and a 2D fast Fourier transform was performed to obtain the spatial frequency spectrum. A 2D high-pass filter with a radial frequency response and half-power cutoff frequency of 1.23 mm^−1^ was applied to suppress low spatial frequencies within the kernel. To increase spatial frequency resolution, the filtered kernels were then zero-padded to 128 × 128 pixels in size. For each trial, the average distance of the peak spatial frequency (PSF) from the spectral origin in the frequency spectrum of all kernels was used as an estimate of the packing density and alignment of the collagen bundles^47^, and the average of the two recordings was used in the analysis. Low values of PSF translate to a less condensed and more isotropic speckle pattern in the ultrasound images, which may serve as an indication for potentially degenerative collagen disorganization within the tissue^17,47^.

### Statistics

A one-way analysis of variance (ANOVA) with Bonferroni post-hoc tests was conducted in SPSS (IBM SPSS Statistics, Version 25, SPSS Inc., Chicago, IL, USA) to examine differences between the participants in each protocol and the control group considering anthropometry. All other outcome parameters (i.e., plantarflexion moment during MVC, AT force, normalized stiffness, AT rest length, strain, lever arm, distal and mid-portion PSF of the free AT and free AT length, CSA, and stress) were analyzed for effects of time, protocol, and their interaction using linear mixed-effects models fitted with restricted maximum likelihood estimation processed with the *nlme* package in R (version 4.1.2; R Core Team 2021). These models are capable of handling missing data and robust to violations of the normality assumption^48^, which, according to the Shapiro–Wilk test, could not be assumed for the normalized residuals of MVC, maximum AT force, lever arm, normalized stiffness, and distal-portion PSF and length of the free AT. Different error variances per protocol were modelled when the variance homogeneity assumption was violated. In case of significant effects of time or time-by-protocol interaction, post-hoc comparisons between the different time points (week 0–week 8; week 8–week 16; week 0–week 16) were performed for each protocol using the *emmeans* package. The corresponding p-values were adjusted according to the method proposed by Benjamini–Hochberg^49^. The alpha-level for all tests was set to 0.05. For the main outcome parameters, Cohen’s *d* was calculated as an effect size for the changes between baseline and week 16 using the pooled standard deviation^50^. Inferences with regard to differences in the effectiveness of the loading protocols were made based on the 95% confidence intervals of these effect sizes.

## Results

The anthropometric data of the participants assigned to the respective protocols and the control group are shown in Table 2. The one-way ANOVA indicated no significant differences in age (*p* = 0.428), body height (*p* = 0.616), mass (*p* = 0.182), and physical activity level (*p* = 0.650) between groups.

**Table 2:** Anthropometric data of the participants.

	HFHV n = 12	HFLV n = 11	LFHV n = 12	LFLV n = 13	REF n = 11	Control n = 13
Age (years)	28.0 ± 6.8	25.1 ± 6.0	27.9 ± 5.8	25.8 ± 5.4	25.0 ± 5.2	28.8 ± 5.0
Height (cm)	179.6 ± 7.1	180.5 ± 7.2	181.1 ± 6.7	183.6 ± 7.4	181.5 ± 8.5	178.3 ± 8.0
Mass (kg)	75.8 ± 7.6	73.9 ± 7.9	80.6 ± 10.0	82.7 ± 11.1	74.9 ± 8.6	76.3 ± 10.2
Physical activity (hours/week)	6.5 ± 3.4	8.2 ± 4.4	7.0 ± 4.3	8.3 ± 4.6	6.0 ± 3.6	6.7 ± 3.2

Physical activity refers to the weekly hours of vigorous physical activity (e.g. recreational training).

HFHV, high frequency, high volume; HFLV, high frequency, low volume; LFHV, low frequency, high volume; LFLV, low frequency, low volume; REF, reference protocol.

**Figure 3:**
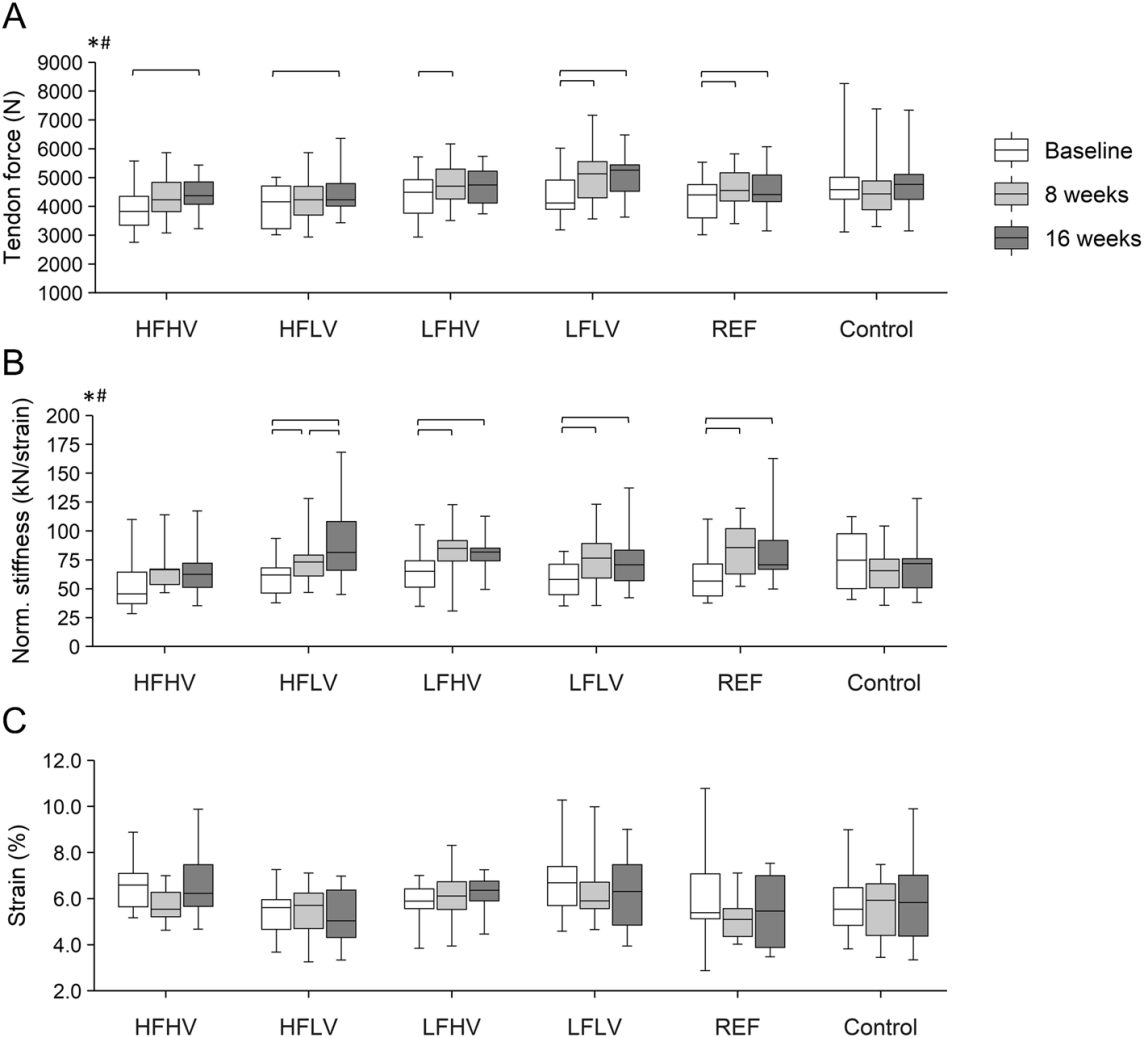
(**A**) Maximum force applied to the Achilles tendon, (**B**) normalized stiffness, and (**C**) strain in the participants of the five intervention protocols and the control group. HFHV, high frequency, high volume (n = 12); HFLV, high frequency, low volume (n = 11); LFHV, low frequency, high volume (n = 12); LFLV, low frequency, low volume (n = 13); REF, reference protocol (n = 11); Control: n = 13. ^*^significant main effect of time; ^#^significant interaction of time by protocol; brackets indicate significant post-hoc differences (*p* < 0.05).

The linear mixed-effects model showed a significant effect of time on the maximum force applied to the AT and normalized AT stiffness (both *p* < 0.001) with time-by-protocol interaction (force: *p* < 0.001; stiffness: *p* = 0.013; Fig. 3A,B). The post-hoc tests demonstrated a significant increase in AT force in the LFHV, LFLV and REF protocols after 8 weeks (*p* = 0.042, < 0.001, and = 0.042, respectively) and in all loading protocols except LFHV after 16 weeks (HFHV and HFLV: p = 0.042; LFLV: *p* < 0.001; REF: *p* = 0.048; Fig. 3A). The increase of normalized AT stiffness was significant after 8 weeks following all loading protocols (HFLV: *p* = 0.049; LFHV: *p* = 0.008; LFLV: *p* = 0.021; REF: *p* = 0.008) except HFHV (*p* = 0.082; Fig. 3B). Both, the maximum force applied to the AT as well as normalized AT stiffness did not show significant changes in the control group (force: *p* = 0.958; stiffness: *p* = 0.661). In all groups, maximum strain (Fig. 3C) did not change significantly over time (time: *p* = 0.474; interaction: *p* = 0.234).

**Figure 4:**
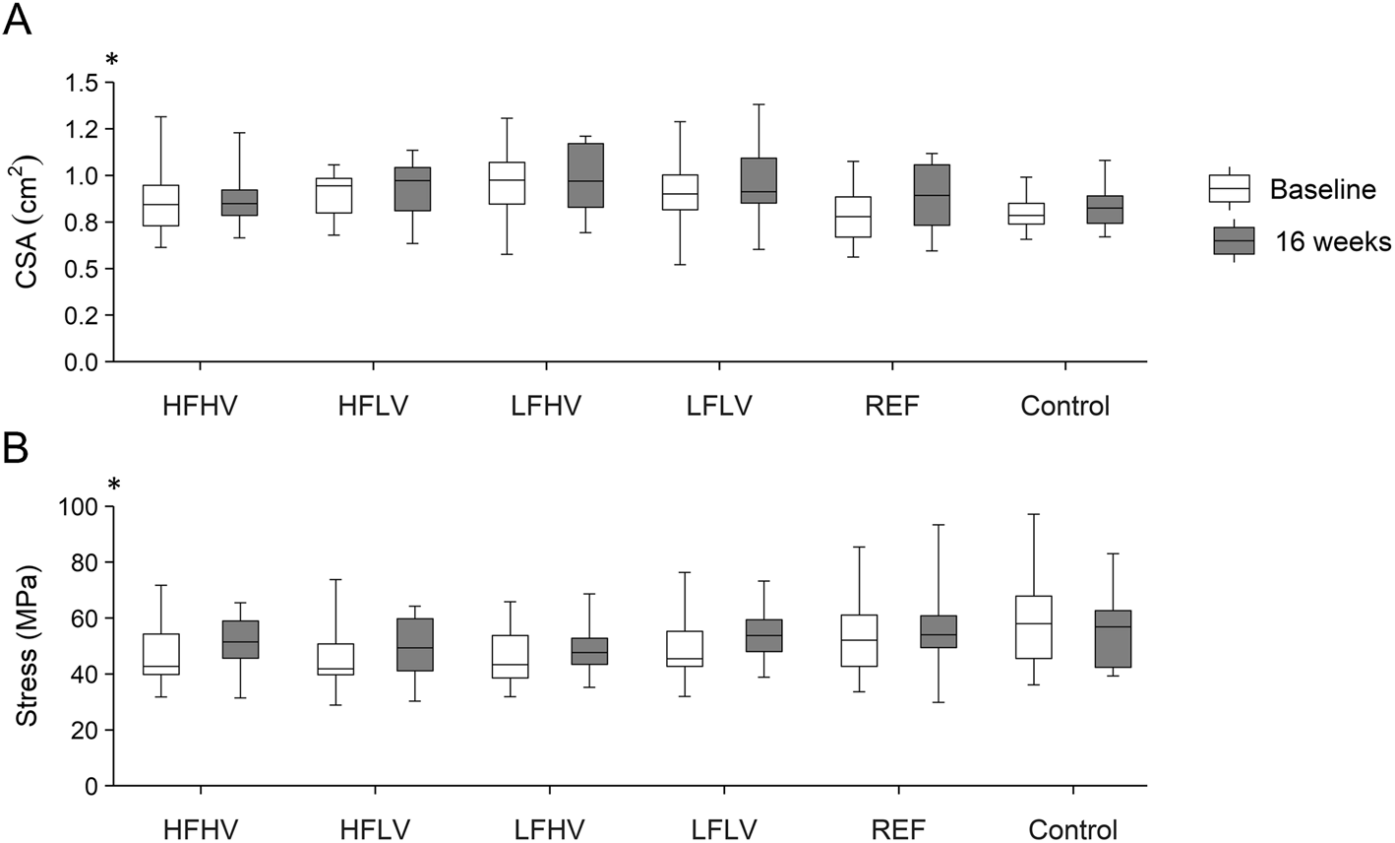
(**A**) Average free Achilles tendon cross-sectional area (CSA) and (**B**) maximum free Achilles tendon stress in the participants of the five intervention protocols and the control group. HFHV, high frequency, high volume (n = 12); HFLV, high frequency, low volume (n = 11); LFHV, low frequency, high volume (n = 12); LFLV, low frequency, low volume (n = 13); REF, reference protocol (n = 11); Control: n = 13. ^*^significant main effect of time.

There was a significant effect of time on the average CSA and stress of the free AT (*p* = 0.003 and 0.023, respectively), indicating an increase without time-by-protocol interaction (*p* = 0.675 and 0.499; Fig. 4A,B). However, the single post-hoc comparisons did not reach the significant level (CSA: HFHV, HFLV, LFHV, and control: *p* = 0.526, LFLV and REF: *p* = 0.100; stress: HFHV, HFLV, and LFLV: *p* = 0.246, LFHV and control: *p* = 0.484, REF: *p* = 0.503).

There was a significant effect of time (*p* < 0.001) on the maximum plantarflexion moment with a significant time-by-protocol interaction (*p* < 0.001; Table 3). The increase was significant following the LFHV, LFLV and REF protocols after 8 weeks (*p* = 0.046, *p* < 0.001, and *p* = 0.046, respectively) and following all loading protocols except LFHV (*p* = 0.228) after 16 weeks (HFHV and HFLV: *p* = 0.046; LFLV: *p* < 0.001; REF: *p* = 0.049), while there were no significant changes in the control group (after 8 weeks: *p* = 0.980; after 16 weeks: *p* = 0.947; Table 3). The lever arm of the AT did not show a significant effect of time (*p* = 0.983) or time-by-protocol interaction (*p* = 0.150; Table 3). There was a time-effect (*p* = 0.039) on the AT rest length (measured to the GM-MTJ), nevertheless, the post-hoc comparisons did not show any significant changes within the protocols (Table 3). Further, there was no significant effect of time (*p* = 0.374) or time-by-protocol interaction (*p* = 0.168) on the rest length of the free AT determined based on MRI (Table 3). The distal free AT PSF showed a significant time-by-protocol interaction (*p* = 0.011), whereas no significant time-effect (*p* = 0.776) or time-by-protocol interaction (*p* = 0.792) was found considering the mid-portion. The post-hoc comparisons suggested that there was a transient increase in distal free AT PSF in response to the LFHV protocol in week 8 (*p* = 0.013; Table 3).

**Table 3:** Maximum plantarflexion moment (MVC), Achilles tendon (AT) lever arm, AT rest length, free AT length, and distal- and mid-portion peak spatial frequency (PSF) of the free AT in the participants of the five intervention protocols and the control group.

		HFHV n = 12	HFLV n = 11	LFHV n = 12	LFLV n = 13	REF n = 11	Control n = 13
MVC (Nm)*^#^	Baseline	230 ± 50.4^b,c^	224 ± 45.3^c^	253 ± 53.6^b^	250 ± 41.0^b,c^	230 ± 43.0^b,c^	245 ± 52.2
	Week 8	252 ± 55.3^a^	239 ± 47.5	278 ± 47.9^a^	286 ± 43.1^a^	253 ± 42.6^a^	247 ± 50.2
	Week 16	258 ± 43.4^a^	249 ± 45.9^a^	268 ± 29	286 ± 40.9^a^	244 ± 42.5^a^	246 ± 42.8
Lever arm (mm)	Baseline	59.2 ± 10.0	56.5 ± 4.9	59.3 ± 7.0	53.1 ± 6.7	55.7 ± 5.5	53.5 ± 6.0
	Week 8	57.8 ± 10.0	54.9 ± 4.1	56.6 ± 8.4	59.2 ± 6.3	53.5 ± 9.4	55.7 ± 7.1
	Week 16	58.8 ± 6.8	56.9 ± 8.1	57.4 ± 6.7	58.7 ± 6.2	55.2 ± 8.3	50.1 ± 6.5
AT rest length (mm)*	Baseline	193 ± 30	206 ± 32	197 ± 21	191 ± 21	204 ± 20	199 ± 31
	Week 8	181 ± 21	199 ± 25	191 ± 20	190 ± 25	205 ± 13	199 ± 23
	Week 16	192 ± 26	199 ± 25	198 ± 20	198 ± 24	199 ± 21	188 ± 30
Free AT length (mm)	Baseline	44.4 ± 13.5	43.2 ± 15.1	37.8 ± 19.3	50.9 ± 20.7	53.3 ± 23.2	52.6 ± 16.9
	Week 16	43.2 ± 13.7	44.0 ± 14.5	37.8 ± 18.6	51.1 ± 20.4	54.5 ± 25.1	53.3 ± 17.0
Distal-portion PSF (mm^−1^)*	Baseline	1.70 ± 0.19	1.72 ± 0.15	1.64 ± 0.14^b^	1.81 ± 0.20	1.80 ± 0.12	N/A
	Week 8	1.75 ± 0.07	1.75 ± 0.19	1.84 ± 0.16^a^	1.77 ± 0.11	1.68 ± 0.13	N/A
	Week 16	1.79 ± 0.12	1.70 ± 0.18	1.77 ± 0.10	1.76 ± 0.11	1.72 ± 0.12	N/A
Mid-portion PSF (mm^−1^)	Baseline	1.77 ± 0.12	1.78 ± 0.09	1.75 ± 0.14	1.75 ± 0.16	1.80 ± 0.10	N/A
	Week 8	1.73 ± 0.13	1.74 ± 0.13	1.75 ± 0.13	1.77 ± 0.11	1.77 ± 0.23	N/A
	Week 16	1.81 ± 0.13	1.72 ± 0.17	1.71 ± 0.16	1.76 ± 0.15	1.81 ± 0.13	N/A

HFHV, high frequency, high volume; HFLV, high frequency, low volume; LFHV, low frequency, high volume; LFLV, low frequency, low volume; REF, reference protocol; N/A, not applicable.

^a,b,c^significant difference from ^a^baseline, ^b^week 8, ^c^week 16; *p* < 0.05.

*Significant main effect of time.

^#^Significant interaction of time by protocol.

The confidence intervals of the effect sizes did not indicate clear differences between the loading protocols with regard to pre-post changes on the maximum plantarflexion moment, maximum force applied to the AT during the MVC, normalized stiffness, tendon CSA or stress (Table 4).

**Table 4:** Effect size Cohen’s d and respective lower and upper confidence interval (brackets) for the changes from baseline to week 16 in the maximum plantarflexion moment (MVC), maximum force applied to the Achilles tendon, normalized stiffness, average free Achilles tendon cross-sectional area (CSA) and maximum free Achilles tendon stress.

	HFHV n = 12	HFLV n = 11	LFHV n = 12	LFLV n = 13	REF n = 11	Control n = 13
MVC	0.61 [− 0.02, 0.98]	0.55 [0.09, 1.00]	0.34 [− 0.09, 0.77]	0.90 [0.53, 1.27]	0.49 [0.06, 0.91]	0.02 [− 0.15, 0.18]
Tendon force	0.52 [0.09, 0.94]	0.50 [0.09, 0.90]	0.33 [− 0.04, 0.71]	0.83 [0.49, 1.16]	0.46 [0.07, 0.85]	0.00 [− 0.17, 0.18]
Norm. stiffness	0.49 [− 0.01, 0.99]	1.31 [0.73, 1.89]	0.61 [0.11, 1.11]	0.85 [0.33, 1.38]	0.90 [0.34, 1.45]	− 0.12 [− 0.6, 0.35]
CSA	0.09 [− 0.20, 0.38]	0.12 [− 0.18, 0.43]	0.09 [− 0.20, 0.38]	0.33 [0.04, 0.63]	0.36 [0.02, 0.70]	0.16 [− 0.17, 0.50]
Stress	0.34 [− 0.06, 0.74]	0.32 [− 0.09, 0.74]	0.17 [− 0.23, 0.57]	0.31 [− 0.09, 0.71]	0.15 [− 0.30, 0.61]	− 0.21 [− 0.67, 0.25]

HFHV, high frequency, high volume; HFLV, high frequency, low volume; LFHV, low frequency, high volume; LFLV, low frequency, low volume; REF, reference protocol.

## Discussion

The present study investigated the effects of the temporal coordination of loading and recovery and loading volume on the time course of Achilles tendon adaptation. Four loading protocols were defined, which differed either in the training frequency per week (2.5 or 5 times per week) or loading volume, modulated via the number of sets and resulting in a weekly time under loading of 180 or 300 s at a high loading intensity (i.e., 90% MVC). An additional loading protocol with intermediate training frequency (4 times per week) and volume (240 s of weekly time under loading), which has consistently been found to introduce AT adaptation^24–26,51^, was integrated as a reference protocol. Against our hypothesis, the temporal coordination of loading and recovery did not notably affect the intervention outcomes. Similarly, no clear effects of overall loading volume were observed. The major changes in normalized tendon stiffness occurred already within the first eight weeks, and no further significant changes were found thereafter.

It is well established that tendons can adapt to mechanical loading and show an increase in stiffness, given a sufficient magnitude of tendon strain^33,52^. The loading protocol referred to as the reference protocol in the present study, consisting of five sets of four isometric contractions each at 90% MVC, showed an increase in tendon stiffness of 36 to 54% in previous studies^24–26^. The strain on the tendon during exercise in these studies was approximately 4.5 to 6.5%. In the present study, the loading protocols were applied using a self-developed home-training system. In a subgroup of 18 participants, the Achilles tendon strain during an MVC in the home-training system was measured at the baseline assessment and was on average 6.8 ± 1.3%. Thus, the strain during training at 90% MVC should have been comparable to our previous studies (i.e., 4.5 to 6.5%). Accordingly, with an increase in normalized tendon stiffness of 34%, the reference protocol in the present study showed similar effects to those reported earlier^24–26^. However, to our knowledge, this is the first experimental study in which the temporal coordination of loading and recovery and the total loading volume were systematically modulated. A significant time-protocol interaction was found in normalized stiffness and while no significant changes were observed in the control group, normalized stiffness increased following most protocols, indicating no systematic differences between high, intermediate (i.e., REF) and low-frequency loading. Based on current evidence, one may expect that an acute bout of mechanical loading leads to a net anabolic response of the tendon between 24 and 48 h after cessation^29^. Yet, providing consecutive bouts of loading to the tissue when it attained its highest net-anabolic state apparently does not lead to greater overall responses compared with longer rest periods between sessions when the total (weekly) loading volume is similar. Under the assumption that the dynamic behavior of anabolic and catabolic processes described above generally holds true in consecutive bouts of loading, our results suggest that the efficacy of repeated mechanical stimuli seems rather independent of the current metabolic state of the tendon.

Against our expectations, there was also no clear effect of weekly loading volume on the adaptation of Achilles tendon mechanical properties and the low volume protocols were at least similarly effective compared to intermediate- and high-volume loading. It has been demonstrated in bone adaptation^53^ and suggested for the synthesis of tendon extracellular matrix components as well, that the acute loading responses may demonstrate a “ceiling effect” after a comparatively low loading volume^29^. In vitro data from Paxton and colleagues^23^ suggest that after a certain time of loading, tenocytes are refractory with respect to renewed loading-induced collagen synthesis for a period of time. In their experiments, tendon cells stretched at 1 Hz for up to 60 min showed a significant increase in their anabolic activity in the first 10 min and subsequent decline. Therefore, low-volume loading programs may elicit similar net metabolic responses as intermediate or high-volume loading, thus being more time-efficient and attractive for the integration in the practical field.

It is interesting to note that—at the descriptive level—the high loading volume protocols were associated with comparatively smaller average changes in normalized tendon stiffness in our sample (i.e., small to medium effects), particularly in combination with a high loading frequency. The confidence intervals of the effect sizes demonstrated substantial overlap, which precludes any firm conclusions. However, a linear combination of the protocol-specific coefficients of the linear mixed model enables the comparison of both high and low volume protocols combined (i.e., HFHV + LFHV vs. HFLV + LFLV). The comparison showed significantly greater improvements in normalized stiffness in the low volume protocols (*p* = 0.04). Acute loading of tendons stimulates both anabolic as well as catabolic pathways. It seems possible that an increase in loading volume increases sub-rupture damage progression^54^ and associated catabolic responses^55^, which could lead to a reduction of net protein synthesis if the anabolic response becomes saturated already at lower levels of loading volume. However, our PSF analysis did not suggest substantial effects of the loading protocols on tendon micromorphology and thus no indications for gradual overuse was found neither at the high or lower levels of loading applied in the presenst study.

Essentially, two mechanisms can contribute to a training-induced increase in normalized tendon stiffness. Changes in the material properties of the tendon, for example, initiated by an adjustment in tissue composition or collagen cross-linking, can increase its elastic modulus, the intrinsic resistance of the tissue^56^. In addition, especially in the long-term, morphological changes in terms of hypertrophy can influence the stiffness of tendons^25,26,57,58^. In the present study, we found an average increase in AT cross-sectional area over all loading protocols of 4.3% following 16 weeks of loading. This magnitude is comparable to the effects observed in our previous experimental studies^24–26^, yet clearly smaller compared with the increase in normalized AT stiffness (~ 33% over all protocols). This suggests that the increase in stiffness up to 16 weeks of training is predominantly mediated by modifications of the material properties of the tendon. Further, by integrating an interim examination, the present study was able to differentiate short- and medium-term adaptation effects of the loading protocols. Interestingly, the major changes in tendon normalized stiffness occurred within the first 8 weeks, which included a 3-week habituation period with reduced loading. Although it may be assumed that in principle, a longer duration of an intervention leads to stronger tendon adaptation effects^52^, tendons seem to show a clear adaptation of mechanical properties already after eight weeks, when stimulated appropriately. The collagen turnover of adult tendons is considered relatively low^59^, despite a notably increased collagen synthesis in response to loading^60^. However, the turnover of non-collagenous matrix components is quite high^61^ and an increase in cross-linking at the different hierarchical levels of the collagenous structures (i.e., fascicles, fibers, fibrils, collagen triple helix) could already affect the material properties of tendons in the short term^56^. Accordingly, an upregulation of cross-linking enzymes has been observed in response to acute loading in animal experiments^22,62^. There are now several reports from intervention studies in humans that tendon stiffness can increase after as little as four to six weeks of targeted loading^63–65^ and the disproportionate increase of normalized AT stiffness compared to the AT CSA indicates that modifications of tendon material properties are likely the basis for such short-term adaptation phenomena.

Within all loading protocols, there was a considerable variation of the pre-to-post changes of tendon stiffness (range of CVs from 76.5 to 126.0%), which suggests that individual factors are quite important for the responsiveness to mechanical loading. For example, the individual genetic disposition influences the activity of the mechanosensitive ion channel PIEZO1, which contributes to the translation of mechanical loading into a cellular calcium ion response and the associated regulation of cross-linking enzymes^22^. In addition, data on gene expression and metabolic activity of tendons from healthy individuals and patients with tendinopathy also suggest possible individual differences in tissue turnover, even in the absence of clinical symptoms^66,67^. Furthermore, in the present study, as in all previous studies on tendon adaptation in vivo, loading during training was regulated based on the ankle joint moment received during the MVCs. The strain values measured during an isometric MVC in the home-training system ranged from 4.6 to 8.9%, which shows that the actual mechanical strain on the tendon during the loading protocols was not uniform despite a standardized training intensity of 90% MVC. Since the magnitude of tendon strain and the associated tendon cell deformation significantly influence the metabolic response and adaptation of tendon mechanical properties^25,68,69^, the lack of control of tendon strain during training likely contributes to the individual variability in the training response. It seems likely that a greater homogeneity of training effects could be achieved by individualizing the training load and standardizing the actual strain during training within the range that is currently considered particularly effective^70^.

In the present study, a linear mixed model was used for inferential statistics, as these models have the advantage of being able to handle missing data and are also robust to non-normal error distributions^48,71^. However, as the presence of outliers can challenge the robustness of these models^72^, we used Dixon’s Q test to identify and exclude outliers in our main outcome parameters and were able to confirm our findings and conclusions (see Table 1, Supplementary material).

In conclusion, the present study provides first experimental evidence that the adaptation of human Achilles tendon mechanical properties is rather independent of the temporal coordination of loading and recovery and can be effectively stimulated with a comparatively low loading volume (i.e., 180 s per week at 90% MVC). An increase in loading volume does not seem to further promote tendon adaptation, which indicates a “ceiling” effect in the acute responses to loading and makes low-volume loading programs (e.g., fifteen sets per week of 4 × 3 s loading at 4.5 to 6.5% strain) the more time-efficient and attractive approach for tendon adaptation. Furthermore, the main changes in Achilles tendon stiffness occurred within the first 8 weeks and were most likely mediated by changes in the material properties. While it is yet unclear if these findings transfer to other cohorts (e.g., women, other age groups or patients), they have important implications for the design of loading programs in the field of sports, rehabilitation, and injury prevention.

### Supplementary Information

Supplementary Table 1.

## Data Availability

The source data are available to verified researchers upon request by contacting the corresponding author.
