# Author Correction: Effect of the temporal coordination and volume of cyclic mechanical loading on human Achilles tendon adaptation in men

**DOI:** 10.1038/s41598-024-61081-8

**Published:** 2024-05-06

**Authors:** Meng-Shiuan Tsai, Theresa Domroes, Nikolaos Pentidis, Sophia Koschinski, Arno Schroll, Sebastian Bohm, Adamantios Arampatzis, Falk Mersmann

**Affiliations:** 1 Department of Training and Movement Sciences, Humboldt-Universität zu Berlin, Berlin, Germany; 2 Berlin School of Movement Science, Berlin, Germany

Correction to: *Scientific Reports* 10.1038/s41598-024-56840-6, published online 22 March 2024

The original version of this Article contained errors in Table 1, where the data for “Duration between sessions (h)” for groups “LFHV”, “LFLV” and “REF” was incorrect. The correct and incorrect data appears below.

Incorrect:

**Table:** 

Group	Duration between sessions (h)
LFHV	48–144
LFLV	48–144
REF	24–96

Correct:

**Table:** 

Group	Duration between sessions (h)
LFHV	48–96
LFLV	48–96
REF	24–72

The original Article has been corrected.

