# Supplementary material for: Effect of the temporal coordination and volume of cyclic mechanical loading on human Achilles tendon adaptation in men
Source: Sci Rep. 2024 Mar 22;14:6875. doi: 10.1038/s41598-024-56840-6 (PMC10960029; doi:10.1038/s41598-024-56840-6)
Supplement: Supplementary file 1 — Supplementary Table 1. [file 41598_2024_56840_MOESM1_ESM.docx]

Supplementary material

***Table 1. False discovery rate adjusted p-values [and outlier counts] of key parameters (maximum force applied to the Achilles tendon, normalized stiffness, and strain) excluding outliers with Dixon method in the five intervention protocols and the control group***

|  | Main effects of protocol or time or interaction |  | HFHV n = 12 | HFLV n = 11 | LFHV n = 12 | LFLV  n = 13 | REF  n = 11 | Control n = 13 |
| --- | --- | --- | --- | --- | --- | --- | --- | --- |
| Tendon force^*#^ | Protocol: p = 0.293  Time: p < 0.001  Time-by-protocol: p < 0.001 | *wk_0_-wk_8_* | 0.134 | 0.243 | 0.042 | 0.000 | 0.042 | 0.193 |
|  |  | *Outlier* | [0] | [0] | [0] | [0] | [0] | [1H] |
|  |  | *wk_8_-wk_16_* | 0.611 | 0.380 | 0.491 | 0.631 | 0.974 | 0.974 |
|  |  | *Outlier* | [0] | [0] | [0] | [0] | [0] | [0] |
|  |  | *wk_0_-wk_16_* | 0.042 | 0.042 | 0.134 | 0.000 | 0.049 | 0.131 |
|  |  | *Outlier* | [0] | [0] | [0] | [0] | [0] | [1H] |
| Normalized stiffness^*#^ | Protocol: p = 0.632  Time: p < 0.001  Time-by-protocol: p = 0.012 | *wk_0_-wk_8_* | 0.111 | 0.047 | 0.010 | 0.023 | 0.009 | 0.644 |
|  |  | *Outlier* | [0] | [0] | [0] | [0] | [0] | [0] |
|  |  | *wk_8_-wk_16_* | 0.979 | 0.033 | 0.644 | 0.644 | 0.644 | 0.825 |
|  |  | *Outlier* | [1H] | [0] | [0] | [0] | [0] | [0] |
|  |  | *wk_0_-wk_16_* | 0.087 | 0.000 | 0.033 | 0.008 | 0.033 | 0.677 |
|  |  | *Outlier* | [0] | [0] | [0] | [0] | [1H] | [0] |
| Strain | Protocol: p = 0.079  Time: p = 0.450  Time-by-protocol: p = 0.242 | *wk_0_-wk_8_* | N/A | | | | | |
|  |  | *Outlier* | [0] | [0] | [0] | [0] | [0] | [1H] |
|  |  | *wk_8_-wk_16_* | N/A | | | | | |
|  |  | *Outlier* | [0] | [0] | [0] | [0] | [0] | [0] |
|  |  | *wk_0_-wk_16_* | N/A | | | | | |
|  |  | *Outlier* | [0] | [0] | [0] | [0] | [0] | [0] |

*HFHV: high frequency, high volume; HFLV: high frequency, low volume; LFHV: low frequency, high volume; LFLV: low frequency, low volume; REF: reference protocol. ^*^ significant main effect of time; ^#^ significant interaction of time by protocol; p < 0.05. wk: week; wk_0_: baseline. [H]: high outlier. N/A: not applicable.*
